# Bidirectional association between metabolic syndrome and osteoarthritis: a meta-analysis of observational studies

**DOI:** 10.1186/s13098-020-00547-x

**Published:** 2020-05-07

**Authors:** Sheng-Yao Liu, Wen-Ting Zhu, Bin-Wei Chen, Yuan-Hong Chen, Guo-Xin Ni

**Affiliations:** 1 Department of Orthopeadics, The Second Affilicated Hospital of Guangzhou Medical University, Guangzhou, China; 2 Department of Pharmacy, The Third Affiliated Hospital of Guangzhou Medical University, Guangzhou, China; 3 School of Sport Medicine and Rehabilitation, Beijing Sport University, Beijing, China

**Keywords:** Metabolic syndrome, Osteoarthritis, Cohort study, Cross-sectional study, Meta-analysis

## Abstract

**Background:**

Emerging observational studies suggest an association between metabolic syndrome (MetS) and osteoarthritis (OA). This meta-analysis was conducted to examine whether or not there is a bidirectional relationship between MetS and OA.

**Methods:**

The PubMed and Embase databases were searched from their inception to October 2019. We selected studies according to predefined criteria. Random effects were selected to calculate two sets of pooled risk estimates: MetS predicting OA and OA predicting MetS.

**Results:**

A total of seven cross-sectional studies and four cohort studies met the criteria for MetS predicting the onset of OA. Another six cross-sectional studies and one cohort study met the criteria for OA predicting the onset of MetS. The pooled odds risk (OR) for OA incidences associated with baseline MetS was 1.45 (95% CI 1.27–1.66). The OR for MetS incidences associated with baseline OA was 1.90 (95% CI 1.11–3.27). In an overall analysis, we found that MetS was associated with prevalent OA in both cross-sectional studies (OR = 1.32, 95% CI 1.21–1.44) and cohort studies (OR = 1.76, 95% CI 1.29–2.42). No indication of heterogeneity was found in the cross-sectional studies (p = 0.395, I^2^ = 4.8%), whereas substantial heterogeneity was detected in the cohort studies (p = 0.000, I^2^ = 79.3%).

**Conclusion:**

Meta-analysis indicated a bidirectional association between MetS and OA. We advise that patients with MetS should monitor their OA status early and carefully, and vice versa.

## Background

Osteoarthritis (OA) is a major public health problem characterized by joint stiffness, substantial pain and functional limitations in daily activities, contributing to inflammation and gradual deterioration of articular cartilage [1]. Of particular concern is that due to the growing elderly population ascribed to longer life expectancy, the worldwide health and economic burden of OA will likely increase in the future [2]. The pathophysiologic mechanisms of OA are still not precisely understood, but there is a general agreement that biomechanics and excessive mechanical loading of the joint are involved [3]. Some other genetic, metabolic, and neuroendocrine factors may also contribute to increased incidences of OA [4].

Metabolic syndrome (MetS) includes a number of conditions, like abdominal obesity, impaired fasting glucose, hypertension (HTN) and dyslipidaemia (low high-density lipoprotein (HDL) and triglyceridaemia). Its prevalence has increased rapidly [5] and in parallel with the increasing incidences of diabetes and obesity. Previous studies have demonstrated that the risk of cardiovascular disease (CVD) [6] and mortality [7] increase significantly in patients with MetS. It has become a major public health problem and a common clinical condition in countries with a high incidence of obesity and Western dietary patterns.

Recently, a large number of studies have been conducted to investigate the association between MetS and OA since both of them pose significant challenges to public health [8, 9]. However, these results are not consistent. Some studies indicated that MetS is significantly associated with an increased incidence of OA [10–12], whereas many others demonstrated that the incidence of OA is not higher in MetS [13]. A meta-analysis between MetS and OA in 2016 showed that MetS is positively associated with OA of the knee [14]. Recently, many new studies have been conducted to investigate the association between MetS and OA [15–19], and also to compare incidences of MetS between OA and non-OA individuals [20–26]. However, there has yet to be a meta-analysis about the association between baseline OA and MetS. We therefore performed the present meta-analyses with data from observational studies to better understand the bidirectional association between MetS, its components and OA.

## Methods

### Search strategy

The study selection process was performed following the PRISMA (Preferred Reporting Items for Systematic Review and Meta-Analyses) statement. Pubmed and Embase databases were searched from their inception to October 2019. We systematically identified observational studies investigating the association between MetS and OA. “Metabolic syndrome” in combination with “osteoarthritis” was chosen as the primary key search term. In addition, we also manually searched for any references of relevant studies not identified in the database.

### Study selection

Two authors (S.Y. Liu and W.T. Zhu) independently extracted the data from all eligible studies. Discrepancies were resolved by discussion or by a third author (G.X. Ni) for adjudication. The following criteria were adopted for the meta-analysis: (1) study with a population based observational design, (2) published original data relevant to a possible association between MetS and OA, (3) study reporting odds ratio (OR) or relative risk (RR) with 95% confidence interval (CI), and (4) study with data allowing for the calculation of an OR estimate. The latest study was chosen when several similar studies were conducted with overlapping populations. We consulted researchers with professional knowledge concerning unpublished reports to get additional relevant information.

### Statistical analysis

The estimate from each study was used to generate pooled OR using random effects. Two separate analyses were conducted: MetS predicting OA and OA predicting MetS. We used the I^2^ statistic to evaluate heterogeneity. Values of 25%, 50%, and 75% were considered as low, medium, and high heterogeneity respectively. Subgroup analyses were conducted to explore potential variability in the relationships by demographic characteristics. Sensitivity analyses were also performed to evaluate the influence of a single study on the overall effect estimate by omitting one study at a time. We used Begg’s funnel plot and Egger’s test to evaluate publication bias. All statistical analyses were performed using STATA version 12.0. P values were two-sided with a significance level of 0.05.

## Results

### Study selection

**Fig. 1:**
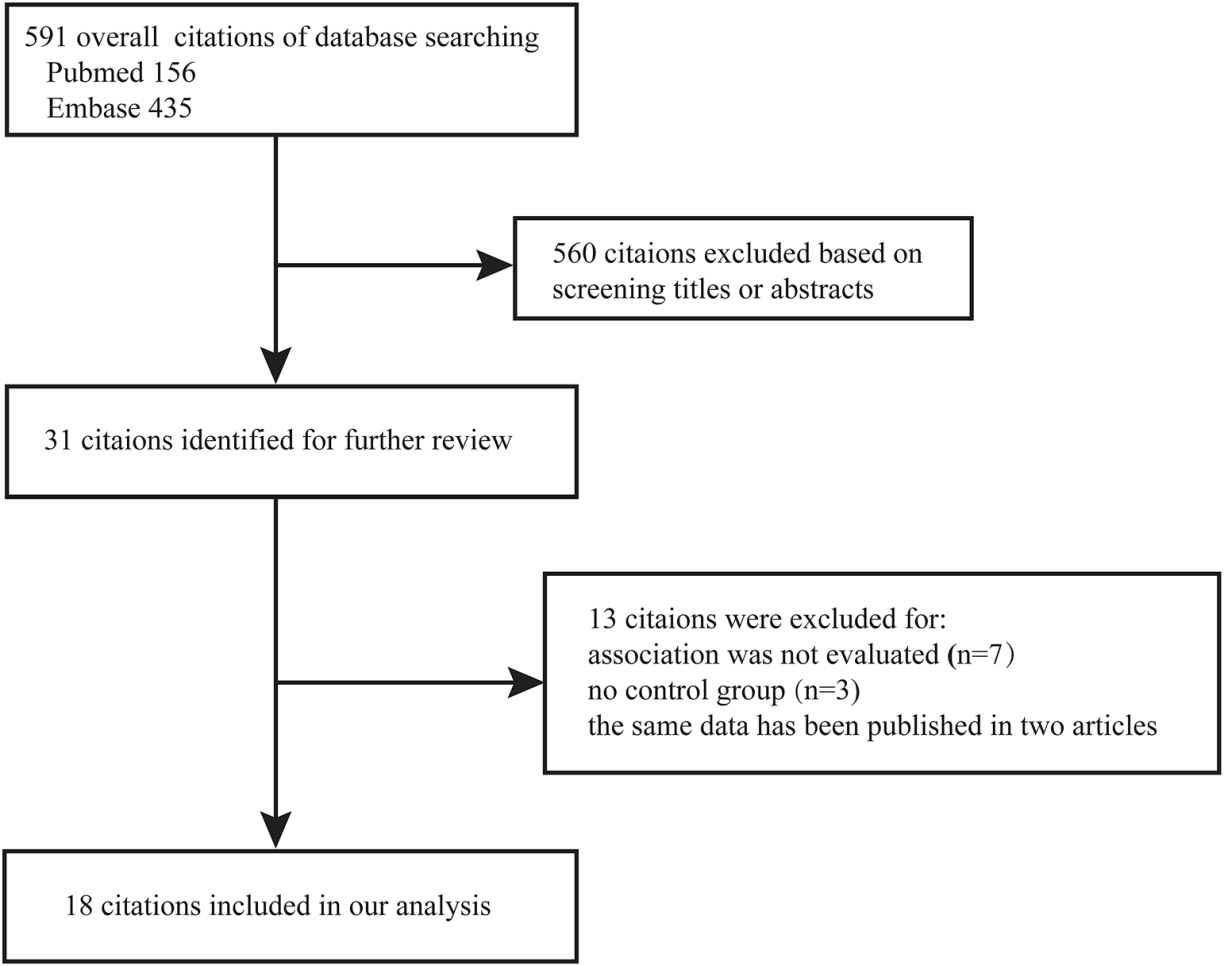
Flow diagram of studies included in the meta-analysis

Figure 1 shows our study selection process. A total of 591 articles were found from two electronic databases. Only 29 articles were left after the first screening based on the aforementioned criteria. After their full texts were then reviewed, 11 more articles were excluded. Seven of them did not investigate the association between MetS and OA [27–33]. Three more did not have a control group [34–36]. Two studies used the same samples and only one was retained [10]. From the remaining articles, 18 (13 cross-sectional studies and five cohort studies) were included for the analysis [10–13, 16–26, 37–39]. Their characteristics are summarized in Tables 1 and 2.

**Table 1:** Characteristics of studies for MetS predicting OA

References	Country	Study design	Number of participants	Age	MetS criteria	OA criteria	Adjustments
Engstrom et al. [38]	Sweden	Prospective cohort study	5171	57.5 ± 5.9	NCEP-ATPIII	Arthroplasty	Age, gender, crp, smoking, physical activity
Yoshimura et al. [10]	Japan	Prospective cohort study	1384	67.3 ± 8.2	Japan Mets criteria	KL ≧ 2 level	Age, gender, region, smoking alcohol, exercise
Monira et al. [11]	Australia	Prospective cohort study	20,430	68.5 ± 7.5	IDF	Joint replacement	Age, gender, ethnic, level of education and physical activity
Han et al. [13]	Korean	Cross-sectional	2234	54.5 ± 11.5	NCEP-ATPIII	Self report	Age, alcohol intake and smoking
Shin et al. [39]	Korean	Cross-sectional	2363	> 50	NCEP-ATPIII	KL ≧ 2 level	Age, gender, income, alcohol consumption physical activity
Eaton et al. [12]	USA	Cross-sectional	212	NR	Modified NCEP-ATPIII	X-ray	NR
Visser et al. [37]	Netherlands	Cross-sectional	6628	56 (50–61)	NCEP ATPIII	X-ray	Age, gender, smoking education, ethnicity
Xie et al. [16]	China	Cross-sectional	5764	55.8 ± 8.0	Modified NCEP-ATPIII	KL ≧ 2 level	Age, sex, activity level, smoking, alcohol and educational background
Niu et al. [17]	USA	Prospective cohort study	991	> 50	NCEP ATPIII	KL ≧ 2 level	Age, education, smoking status, physical activity level
Strand et al. [18]	USA	Prospective cohort study	1089	59.2 ± 6.4	AHA/NHLBI	KL ≧ 2 level	Age and sex
Sanchez et al. [19]	USA	Prospective cohort study	952	54 (49–60)	NCEP ATPIII	KL ≧ 2 level	Age

**Table 2:** Characteristics of studies for OA predicting MetS

References	Country	Study design	Number of participants	Age	MetS criteria	OA criteria	Adjustments
Michishita et al. [21]	NA	Cross-sectional	72	60 ± 6.7	Japan Mets criteria	KL ≧ 2 level	Age
Puenpatom et al. [26]	NA	Cross-sectional	7714	18–90	NCEP ATPIII	X-ray	Age
Inoue et al. [24]	NA	Cross-sectional	795	60 ± 10.4	Modified Japan Mets criteria	KL ≧ 2 level	Age
Maddah et al. [23]	Iranian	Cross-sectional	625	62.4 ± 12.5	NCEP ATPIII	KL ≧ 2 level	Age
Calvet et al. [22]	Spain	Cross-sectional	508	65 ± 8.1	Modified Mets criteria	KL ≧ 2 level	Age
Sakina et al. [20]	Pakistan	Cross-sectional	162	56.6 ± 0.97	AHA MetS criteria	KL ≧ 2 level	Age
Askari et al. [26]	Iranian	Cohort study	393	52.8 ± 8.7	Modified Mets criteria	KL > 1 level	Sex, age

*NA* not available

### Studies of MetS predicting OA risk

The relationship between MetS and the risk of OA was explored in seven cross-sectional studies and four cohort studies. The characteristics of these eleven studies are shown in Table 1. Of these studies, eight identified MetS by the National Cholesterol Education Program (NCEP) Adult Treatment Panel III (ATP III) criteria [12, 13, 16–19, 37–39], one by the International Diabetes Federation (IDF) criteria [11], one by AHA/NHLBI MetS criteria [18] and one by the Japan MetS criteria [10]. OA was identified by X-ray in nine studies [10, 12, 13, 16–19, 37, 39] and knee replacement in two [11, 38]. All included studies enrolled participants aged over 50 [10–13, 16–19, 37–39]. One study was conducted exclusively for women [19] while the others were conducted for both genders, with three studies reporting results separately for men and women [13, 17, 39]. Four studies were conducted in Asia [10, 13, 16, 39], one in Australia [11] and six in Europe [12, 17–19, 37, 38]. The included studies were published between 2009 to 2019, with a total of 40,378 participants. The mean follow-up length ranged from 3 to 12.4 years.

**Fig. 2:**
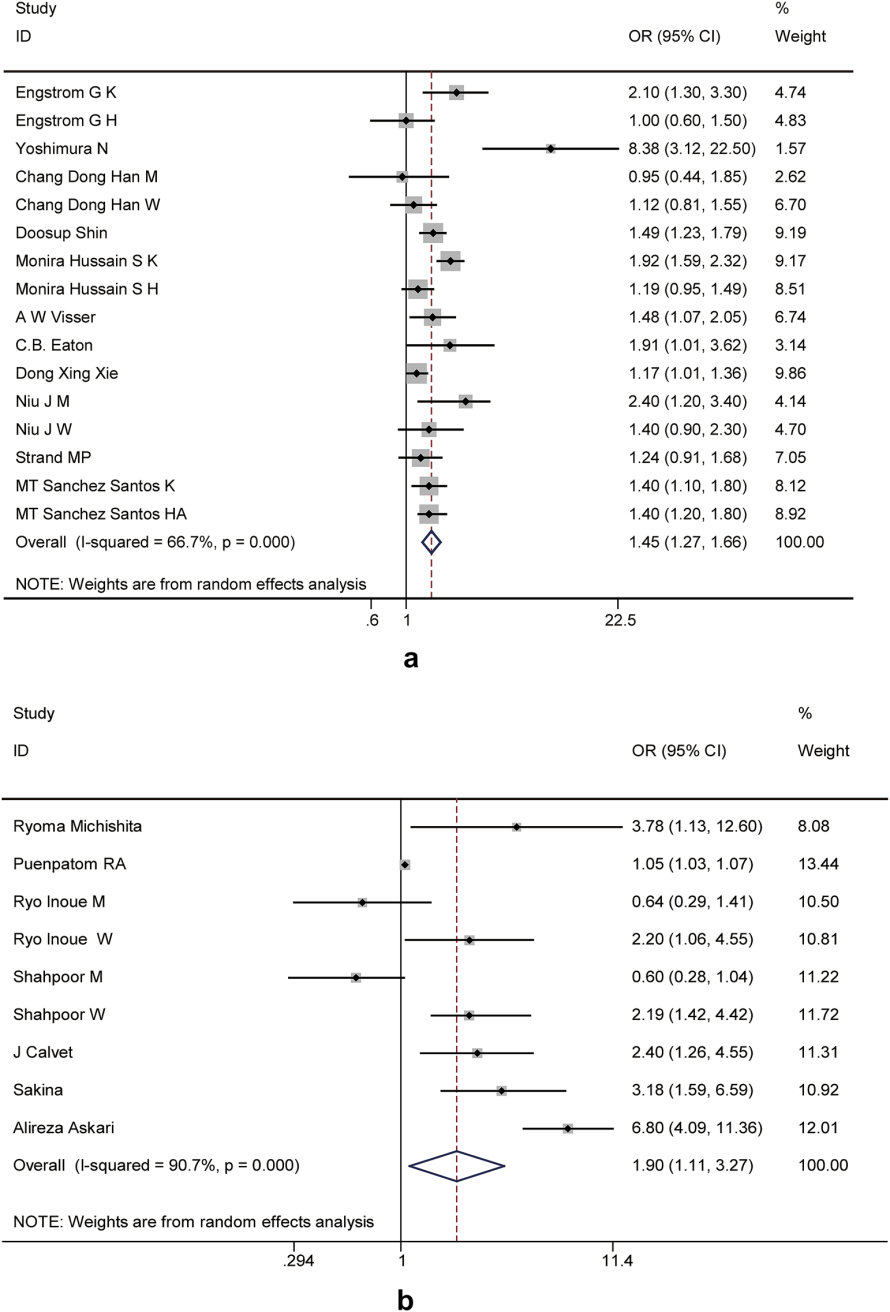
Forest plot of the studies examining the association between MetS and OA. **a** Forest plot for MetS predicting OA; **b** Forest plot for OA predicting MetS. *M* men, *W* women, *K* knee, *H* hip, *HA* hand

**Fig. 3:**
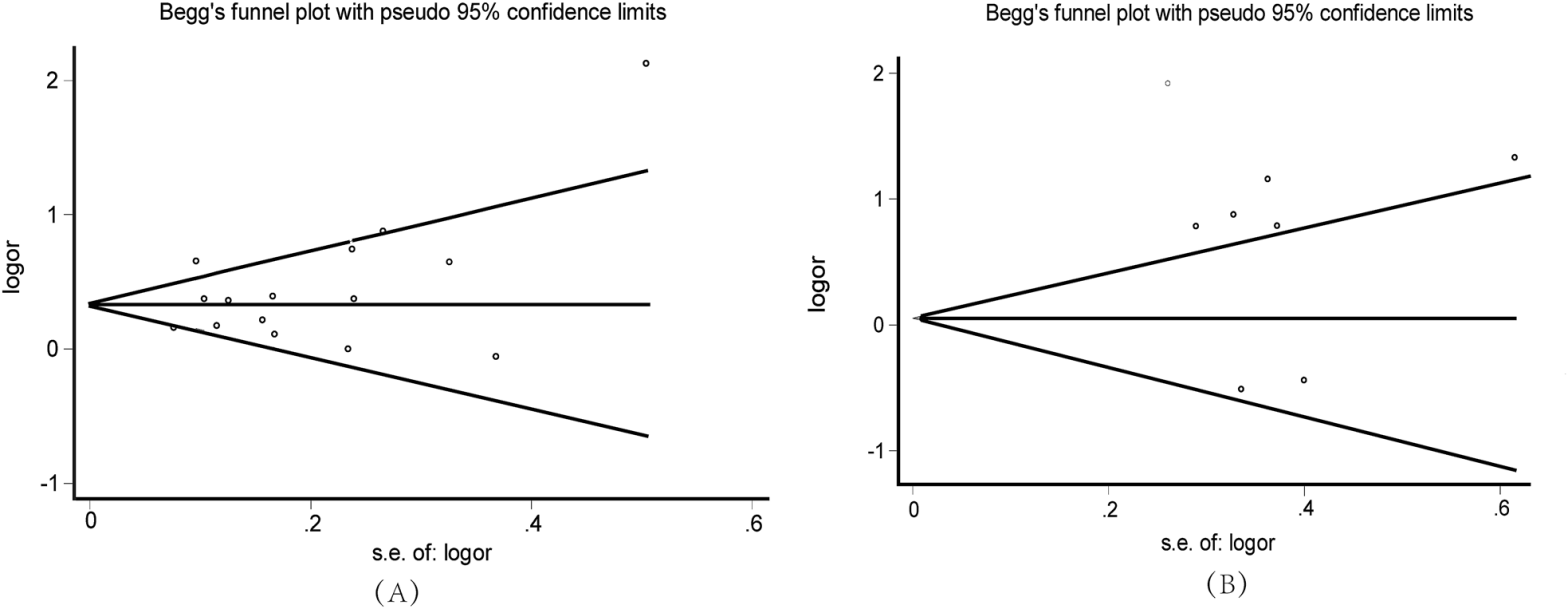
Funnel plot to detect publication bias. **a** Begger’s Funnel plot for MetS predicting OA; **b** Begger’s Funnel plot for OA predicting MetS

OR pooled analyses showed that patients with MetS had a higher overall adjusted risk of OA incidences (OR, 1.45; 95% CI, 1.27–1.66) (Fig. 2a). There was moderate heterogeneity (I^2^ = 66.7%) among the studies, and no publication biases were detected (p = 0.177) (Fig. 3a). Furthermore, when the components of MetS were analyzed separately, OA risk was found to be significantly and positively associated with central obesity (OR, 2.06; 95% CI, 1.71–2.49), hyperglycemia (OA, 1.28; 95% CI, 1.14–1.45), and high blood pressure (OR, 1.27; 95% CI; 1.14–1.42), but not with hypertriglyceridemia (OR, 1.09; 95% CI, 0.95–1.25) and low HDL concentrations (OR, 1.12; 95% CI, 0.96–1.32) respectively. The results of subgroup analyses are shown in Table 3. There was a much stronger association in the cohort study (OR = 1.76 vs 1.32) and European residents (OR = 1.70 vs 1.40) when compared with the cross-sectional study and Asian residents. The association was significant for knee OA (OR, 1.56; 95% CI, 1.31–1.87), hand OA (OR, 1.44; 95% CI, 1.25–1.66) or when using NECP ATP-III criteria to define MetS (OR, 1.38; 95% CI, 1.23–1.56). It was not significant for hip OA (OR, 1.15; 95% CI, 0.94–1.41) or when using IDF MetS criteria (OR, 1.52; 95% CI, 0.92–2.43). Moreover, the association was significant for women (OR, 1.45; 95% CI, 1.10–1.89) but not for men (OR, 1.54; 95% CI, 0.86–2.76) However, since qualified studies in each subgroup were limited, the results should be interpreted cautiously. Sensitivity analysis was performed to assess the effect of each study on the ORs of OA. After excluding those studies without substantially affecting the direction and magnitude of the cumulative estimates, relatively stable results were derived (see Table 4).

**Table 3:** Subgroup analysis of the association between MetS and OA

Subgroup	No of studies	OR (95% CI)	p value heterogeneity	I^2^(%)
MetS predicting OA				
Ethnicity				
Asian	4	1.40 (1.03, 1.90)	0.001	79.1
European	7	1.49 (1.29, 1.71)	0.017	53.7
Study design				
Cohort study	4	1.76 (1.29, 2.42)	0.000	79.3
Cross-sectional study	7	1.32 (1.21, 1.44)	0.395	4.8
Location				
Knee	9	1.56 (1.31, 1.87)	0.000	74.3
Hand	4	1.44 (1.25, 1.66)	0.485	0
Hip	2	1.15 (0.94, 1.41)	0.504	0
MetS criteria				
NCEP ATPIII	8	1.38 (1.23, 1.56)	0.075	39.9
IDF	1	1.52 (0.95, 2.43)	0.001	90.2
OA predicting MetS				
Gender				
Male	3	0.84 (0.61, 1.16)	0.248	28.2
Female	3	2.34 (1.54, 3.56)	0.708	0
MetS criteria				
NCEP ATPIII	2	1.12 (0.65, 1.93)	0.01	78.3
Modified criteria	2	4.12 (1.48, 11.42)	0.901	0
Japan criteria	2	1.64 (0.60, 4.50)	0.020	74.3
Location				
Knee	6	1.55 (1.02, 2.26)	0.000	78.9
Hand	1	2.67 (1.15, 6.19)	0.000	0

**Table 4:** Sensitivity analysis after each study was excluded by turns

Study omitted	OR (95% CI) for remainders	Heterogeneity	
		I^2^ (%)	*p*
a) MetS predicting OA			
Engstrom et al. K	1.42 (1.24, 1.63)	66.7	0.000
Engstrom et al. H	1.48 (1.29, 1.69)	67.4	0.000
Yoshimura et al.	1.41 (1.25, 1.58)	56.8	0.004
Han et al. M	1.47 (1.28, 1.68)	68.1	0.000
Han et al. W	1.48 (1.29, 1.70)	67.5	0.000
Shin et al.	1.45 (1.25, 1.69)	68.6	0.000
Monira et al. K	1.40 (1.23, 1.59)	57.4	0.003
Monira et al. H	1.48 (1.28, 1.71)	67.3	0.000
Visser et al.	1.45 (1.26, 1.67)	68.8	0.000
Eaton et al.	1.44 (1.26, 1.65)	68.3	0.000
Xie et al.	1.49 (1.29, 1.71)	62.9	0.001
Niu et al. M	1.42 (1.24, 1.62)	65.8	0.000
Niu et al. W	1.46 (1.27, 1.67)	68.9	0.000
Strand et al.	1.47 (1.28, 1.69)	68.5	0.000
Sanchez et al. K	1.46 (1.26, 1.69)	68.9	0.000
Sanchez et al. HA	1.46 (1.26, 1.69)	68.9	0.000
b) OA predicting MetS			
Michishita et al.	1.79 (1.02, 3.15)	91.4	0.000
Puenpatom et al.	2.08 (1.13, 3.85)	84.5	0.000
Inoue et al. M	2.16 (1.19, 3.92)	91.7	0.000
Inoue et al. W	1.87 (1.03, 3.38)	91.5	0.000
Maddah et al. M	2.20 (1.20, 4.03)	91.6	0.000
Maddah et al. W	1.87 (1.02, 3.43)	91.2	0.000
Calvet et al.	1.85 (1.02, 3.35)	91.2	0.000
Sakina et al.	1.78 (1.00, 3.17)	90.9	0.000
Askari et al.	1.56 (1.02, 2.39)	79.8	0.000

*K* knee, *H* hip, *HA* hand, *W* women, *M* men

### Studies of OA predicting MetS risk

The relationship between OA and the risk of MetS was investigated in six cross-sectional studies and one cohort study with a total of 10,268 participants. The characteristics of these studies are presented in Table 2. OA was identified by X-ray in all these studies. Of the seven studies, five defined MetS by the NECP ATP-III criteria or its modified version [22–26], one by the Japan MetS criteria [19] and one by the AHA/NHLBI MetS criteria [20]. One study focused on men [25], one on women [21], three others on both genders [20, 22, 26], and two on men and women separately [23, 24].

OR pooled analyses in random-effects models showed that patients with OA had a higher overall adjusted risk of MetS incidences (OR, 1.90; 95% CI, 1.11–3.27) (Fig. 2b) and there was high heterogeneity (I^2^ = 90.7%) among the studies. No publication bias was revealed by Begger’s test (p = 0.677) (Fig. 3b). The subgroup analyses showed significant association in women (OR, 2.34; 95% CI, 1.54–3.56) but not in men (OR, 0.86; 95% CI, 0.61–1.16). The association was more pronounced for hand OA (OR, 2.70 vs 1.55) compared to knee OA. Other stratified variables showed no significant differences. Furthermore, when the components of MetS were analyzed separately, baseline OA had a higher risk for central obesity (OR, 2.87; 95% CI, 2.5–3.29) and knee OA was positively associated with the incidence of hypertension (OR, 2.80; 95% CI, 1.03–7.61). However, as the subgroup number was small, the results should be interpreted cautiously. In addition, sensitivity analyses were conducted by stepwise with one study omitted at a time. The summary ORs of the remaining studies were reevaluated to estimate the impact of a single study on the combined results.

## Discussion

Recently, a number of epidemiological studies, basically cross-sectional and prospective cohort studies, investigated the association between MetS and OA but their results were not completely consistent. To the best of our knowledge, this is the first meta-analysis study to explore the bidirectional associations between MetS and OA. Our results revealed bidirectional associations between MetS and OA in cross-sectional studies and an increased incidence of OA with baseline MetS in prospective cohort studies.

Although many studies reported a higher prevalence of OA in individuals with MetS, they varied considerably in many aspects, including study design, ethnicity, definition of MetS, and participant gender [19, 20]. Therefore, the OR, instead of the prevalence, was pooled. There was a significant effect size in the pooled ORs of studies adjusted for age, gender and ethnicity. The pooled adjusted OR of OA by MetS status was 1.45 (1.27–1.66) and the OR for MetS incidences associated with baseline OA was 1.90 (1.11–3.27).

Our results suggested that the association was much stronger in Western countries than in Asian countries for MetS predicting OA. A possible explanation may be that Western dietary patterns made them more subject to obesity and diabetes, both of which have been proven to be associated with OA [40]. In addition, our results indicated that knee OA rather than hand OA was significantly associated with baseline MetS. It is well-known that both obesity and inflammation contribute to OA and knees are subjected to much greater mechanical loadings than hands, likely leading to OA [41]. What is more, a stronger association was observed in studies defining MetS with the 2005 NCEP ATP-III criterion rather than the 2005 IDF criterion. The only difference between the two criteria is that the 2005 IDF criterion considers central obesity, a strong risk factor of OA, as an obligatory component of MetS [9]. Our results were not consistent with this. On the contrary, our study found that the incidence of MetS was much higher in hand OA individuals than in knee OA individuals. In addition, it seemed that women with OA were more likely to be afflicted by MetS than men. The underlying mechanism should be investigated in the future.

It is well recognized that MetS is a forced combination of multiple components in a single variable. As such, the independent effect of each single component might be influenced. Moreover, selected studies may differ in their definition of MetS and weights of the same factor, thus quite likely generating a non-uniform distribution and hierarchy of the metabolic components. In order to overcome these barriers, the influence of each component was evaluated [12, 13, 39]. The results showed that hypertension, hyperglycemia and abdominal obesity significantly increased the risk of OA. It is suggested that appropriate OA screening should be necessary for individuals with these health conditions no matter whether MetS exists or not. Meanwhile, the risk of obesity and hypertension could be increased in patients with OA.

There are many possible explanations for the association between MetS and OA. First, individuals with MetS have a greater body mass index (BMI) and higher inflammatory cytokines (including interleukin 6 and C-reactive protein, etc.), indicating the physiopathology of OA [38, 42, 43]. Other metabolic disturbances are also associated with OA, including insulin resistance and mitochondrial disorder [44]. Second, as a cluster of vascular risk factors, MetS may lead to subchondral ischaemia. This may cause a reduction in the gas and nutrient exchange between the subchondral bone and articular cartilage, and consequently the development of OA [45, 46]. Furthermore, MetS is positively correlated to a sedentary lifestyle which can increase the incidence of OA. In parallel, OA is also positively associated with obesity, insulin resistance and chronic inflammation, which are also involved in the etiological mechanisms of MetS [47, 48]. Another potential explanation is that OA individuals usually do less amount of exercise than their healthy counterparts, thus making MetS a consequence of OA [49]. Last but not least, medications for OA may affect various components of MetS and partially contribute to such an association [50]. This is one of the main reasons for hand OA predisposing to MetS. Taken together, the potential mechanisms are complex and may involve several shared physiological pathways, such as obesity, inflammation, oxidative stress and mitochondrial disorder. However, in order to better prevent and treat both OA and MetS, further investigations of the potential mechanisms underlying this reciprocal relationship are required.

There are several limitations for this meta-analysis study. First, high heterogeneity resulted from differences in the definition of MetS (including NECP ATP-III, IDF and Japanese criteria), gender, ethnicity, study design, etc. In order to account for the heterogeneity, we chose random-effects models instead of fixed-effects models, but the results were not substantially changed. We conducted subgroup and sensitivity analyses to search for the source of heterogeneity and no significant publish bias was revealed in this meta-analysis. Second, most OA cases in this study were diagnosed by X-ray examination. However, some OA subjects were diagnosed as needing knee replacement, a condition usually more serious than what is revealed by X-ray examination. Therefore, possible misclassification of OA might have biased the results. Third, more cross-sectional studies were selected than cohort ones. A relatively low level of evidence in cross-sectional studies might have limited the quality of this study. Finally, the total number of studies investigating the association between MetS and OA was small. In the future, more prospective cohort studies with a large sample size and various subgroups are needed.

In spite of these limitations, our meta-analysis has yielded significant findings for both public health and clinical care. We demonstrated that MetS is associated with an increased risk of OA and vice versa. Therefore, it is advised that patients with OA should monitor their MetS status early and carefully. If they are at a higher risk of CVD, proper measures and lifestyle modifications should be implemented. What is more, early detection of OA may prevent the incidence of CVD for patients with MetS who are already susceptible to CVD. However, it is not clear whether treatment of MetS/OA can lower the increased risk of OA/MetS. Therefore, well-designed and controlled trials in both animal and human samples are needed in the future.

## Conclusion

In conclusion, the present meta-analysis suggested bidirectional associations between MetS and OA. This serves as a reminder that early and careful monitoring of MetS/OA is crucial for patients with OA/MetS. As limited data are available in this field, more prospective studies should be conducted to better understand the association between MetS and OA. Furthermore, more studies on the underlying mechanisms are warranted in the future.

## Data Availability

Please contact the authors for data requests.
